# The participation of the muscarinic receptors in the preoptic-anterior hypothalamic areas in the regulation of ovulation depends on the ovary

**DOI:** 10.1186/s12958-016-0208-3

**Published:** 2016-11-04

**Authors:** Adriana Espinosa-Valdez, Angélica Flores, Isabel Arrieta-Cruz, Mario Cárdenas, Roberto Chavira, Roberto Domínguez, María Esther Cruz

**Affiliations:** 1 Biology of Reproduction Research Unit, Laboratory of Neuroendocrinology, Facultad de Estudios Superiores Zaragoza, UNAM, AP 9-020, CP 15000 Mexico City, Mexico; 2 Department of Basic Research, National Institute of Geriatrics, México City, Mexico; 3 Instituto Nacional de Ciencias Médicas y Nutrición “Salvador Zubirán”, México City, Mexico

**Keywords:** Unilateral ovariectomy, Ovulation, Diestrus-2, Muscarinic receptors, Atropine, POA-AHA, Estradiol, Gonadotropins

## Abstract

**Background:**

Muscarinic receptors (mAChRs) of the preoptic and anterior hypothalamus areas (POA-AHA) regulate ovulation in an asymmetric manner during the estrous cycle. The aims of the present study were to analyze the effects of a temporal blockade of mAChRs on either side of the POA-AHA performed in diestrus-2 rats on ovulation, the levels of estradiol, follicle stimulating hormone (FSH) and luteinizing hormone (LH) and the mechanisms involved in changes in ovulation.

**Methods:**

Cyclic rats on diestrus-2 day were anesthetized and randomly assigned to the following groups: 1) microinjection of 1 μl of saline or atropine solution (62.5 ng) in the left or right POA-AHA; 2) removal (unilateral ovariectomty, ULO) of the left (L-ULO) or right (R-ULO) ovary, and 3) rats microinjected with atropine into the left or right POA-AHA plus L-ULO or R-ULO. The ovulation rate and the number of ova shed were measured during the predicted estrus, as well as the levels of estradiol, FSH and LH during the predicted proestrus and the effects of injecting synthetic LH-releasing hormone (LHRH) or estradiol benzoate (EB).

**Results:**

Atropine in the left POA-AHA decreased both the ovulation rate and estradiol and LH levels on the afternoon of proestrus, also LHRH or EB injection restored ovulation. L- or R-ULO resulted in a lower ovulation rate and smaller number of ova shed, and only injection of LHRH restored ovulation. EB injection at diestrus-2 restored ovulation in animals with L-ULO only. The levels of estradiol, FSH and LH in rats with L-ULO were higher than in animals with unilateral laparotomy. In the group microinjected with atropine in the left POA-AHA, ovulation was similar to that in ULO rats. In contrast, atropine in the right POA-AHA of ULO rats blocked ovulation, an action that was restored by either LHRH or EB injection.

**Conclusions:**

These results indicated that the removal of a single ovary at noon on diestrus-2 day perturbed the neuronal pathways regulating LH secretion, which was mediated by the muscarinic system connecting the right POA-AHA and the ovaries.

## Background

The preovulatory period of the estrous cycle is characterized by the rapid growth of ovarian follicles and the increased secretion of estradiol (E_2_). Plasma levels of E_2_ are low on estrus day, increasing its levels from the afternoon of diestrus-1 day until the morning of diestrus-2 day, reaching the highest levels between morning and noon on the proestrus day [1]. Ovarian E_2_ secreted on diestrus-2 day is required to activate the LH surge on proestrus day, since the injection of estrogen antagonists [2] or estradiol antiserum [3] or ovariectomy [4] during diestrus-2 day block the preovulatory LH surge.

The LH surge is the result of an increase in the frequency and amplitude of pulses of secretion of gonadotropin-releasing hormone (GnRH) in the POA-AHA, in response to activation of several molecules present in the neural network GnRH [5], such as neurons that synthesize acetylcholine (ACh). Importantly, ACh regulates the secretion of GnRH, LH, and FSH as well as stimulates the weight of ovaries, the ovarian compensatory hypertrophy in ULO of the rats [6–9]. Interestingly, the participation of the muscarinic cholinergic system is relevant to the regulation of ovulation which varies throughout the estrous cycle and has a circadian rhythm [10].

The continuous blockade of mAChRs by implants of atropine crystals on either side of the POA-AHA blocked ovulation depending on the day of the estrous cycle [6]. Similar effects were observed when pilocarpine, a specific agonist of mAChRs, was administered in the POA-AHA [11]. Previous studies performed by our group showed that ULO rats with implants of atropine in the left POA-AHA during the estrus day did not ovulate, while the majority of rats with implants in the right POA-AHA did. Furthermore, the injection of EB restored ovulation in animals with atropine implants in the left POA-AHA [12]. These observations suggested that the cholinergic muscarinic system in the POA-AHA exerted an asymmetric regulating action on spontaneous ovulation through the modutation of GnRH secretion.

The aim of the present study was to examine whether or not the muscarinic system in the POA-AHA participates in regulating ovulation on a permanent or temporary manner. To answer this, we analyzed the effects of ULO on spontaneous ovulation in rats microinjected with atropine into the POA-AHA.

## Methods

### Animals

The study was carried out in 3–4 month old female virgin rats (195–225 g), of the CIIZ-V strain, from our own production stock. The animals were kept under controlled light (05:00–19:00 h) and temperature (22 ± 2 °C) conditions, with free access to tap water and a standard rodent diet (Harlan, SA, Mexico City, Mexico). Estrous cycles were monitored by examining daily vaginal smears, and only animals with at least two consecutive estrous cycles of 4 days each were used in the experiments. All treatments were performed from 12:30 to 13:30 h on diestrus-2 day. After surgery (described below), each animal was placed in a cage with sawdust, warmed with an incandescent lamp until they awoke. After the treatments, vaginal smears were obtained again at 24 h after abdominal or brain surgery. Lastly, the animals were sacrificed at 10:00 h on the predicted estrus.

**Fig. 1:**
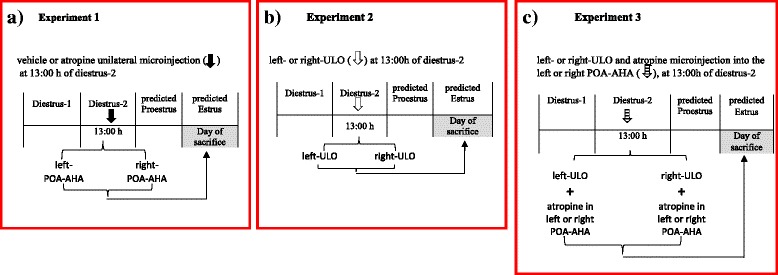
Schematic representation of the experimental design: **a** Experiment 1: Groups of rat’s microinjected with vehicle or atropine in the left or right side of POA-AHA at 13:00 h during diestrus-2. **b** Experiment 2: Rats subjected to removal of the left (left-ULO) or right ovary (right-ULO) at 13:00 h during diestrus-2. **c** Experiment 3: Group of left-ULO or right-ULO rats microinjected with atropine in the left or right POA-AHA at 13:00 h during diestrus-2. All animals were sacrificed on the predicted estrus day

To investigate if the muscarinic system of the POA-AHA is involved in regulating ovulation on a transient or more long-term manner, we designed three different sets of experiments as follows: 1) microinjection of atropine into the left or right POA-AHA; 2) left or right ULO; and 3) left or right ULO, followed of blockade of the mAChRs in the left or right POA-AHA (Fig. 1). Additionally, to investigate the mechanisms affected by blocking mAChRs at 14.00 h of the predicted proestrus day, groups of animals microinjected with atropine in left POA-AHA, left or right ULO or left or right ULO followed by blockade of the mAChRs in the left or right POA-AHA were injected with synthetic LH-releasing hormone (LHRH)-Gly-OH or with estradiol benzoate (EB). Finally, the levels of estradiol, FSH and LH were measured in the morning and afternoon of expected proestrus of animals microinjected with atropine in the left or right POA-AHA or with left or right ULO.

#### Experiment 1. Effects of the transient blockade of mAChRs in the left or right POA-AHA on spontaneous ovulation (Fig. 1A)

To investigate the effects of unilateral transient blockade of mAChRs in the POA-AHA on ovulation, microinjections of atropine (a competitive and reversible antagonist of five mAChRs (m1-m5) [7, 13] were performed in the left or right POA-AHA of rats. The animals were anesthetized with an intraperitoneal injection of sodium pentobarbital (30 mg/kg; Anestesal, Smith-Kline Norden of México City, Mexico) and placed in a stereotaxic apparatus (David Kopff Instruments, Tujunga, CA, USA). An incision in the skin of the dorsal region of the head was made subsequently the left or right side of the skull was exposed and drilled with a stainless steel 1 mm diameter drill. Next, a 29-gauge stainless steel needle was inserted into the left or right POA-AHA. The location of the POA-AHA was determined according to the coordinates (AP 0.650 mm ± 0.06 mm lateral, vertical 0.86 mm) from the König and Klippel Atlas [14]. The microinjection needle was connected through a piece of Teflon tubing (OD 0.65 mm 0.12 mm OI 9; Bioanalytical Systems, Inc., West Lafayette, IN, USA) to a 20-μl Hamilton syringe mounted on a pump microinjector (CMA/100, BAS, Stockholm, Sweden). Rats were randomly assigned to different groups (*n* = 8–10 animals per group) and microinjected as follows: 1) vehicle: saline solution 1 μl; 2) atropine, 62.5 ng/μl (Sigma-Aldrich, Mexico); and 3) intact: cyclically untreated rats. The dose of atropine was selected based on previous studies in our laboratory [6, 12]. All test solutions were injected at a rate of 1 μl/min.

#### Experiment 2. Effects of left or right ULO on spontaneous ovulation (Fig. 1b)

To evaluate the role of each ovary in the physiological mechanisms regulating spontaneous ovulation at 13:00 h of diestrus-2 day, the rats were anesthetized and were randomly assigned to one of the experimental groups described below.

- *Intact Group*: Cyclical rats without treatment, sacrificed at 10:00 h of estrus day.
- *Unilateral Laparotomy*: A unilateral incision was made 1 cm below the last rib, throughout the skin, muscle and peritoneum. The ovaries were left untouched. At the end of the surgical procedure, the wound was sealed.
- *Unilateral Ovariectomy* (ULO): A unilateral incision was made below the last rib, throughout the skin, muscle and peritoneum, and the left or right ovary was carefully removed. The wound was subsequently sealed.

#### Experiment 3. Effects of the transient unilateral blockade of the mAChRs in the POA-AHA region in ULO rats on spontaneous ovulation (Fig. 1c)

To test our hypothesis that the ovary is critical in controlling spontaneous ovulation mediated by the muscarinic system in the hypothalamus, we combined L- or R-ULO with microinjections of atropine in the left or right POA-AHA. The rats were treated as described in experiments 1 and 2. The abdominal and brain surgeries were performed back to back on the same day.

### Postmortem procedures

On the day of expected estrus (46 h after ULO), the rats were sacrificed by decapitation [15], the oviducts were dissected and the number of ova shed was counted using a dissecting microscope (Olympus SZ51-LGB, Tokyo, Japan). Next, the ovaries were removed, dissected and weighed on a precision balance (Mettler, AT-261, Switzerland). The brain was also rapidly dissected and fixed in paraformaldehyde solution (4 % v/v). The ovulation rate (number of animals that ovulated/total number of treated rats) was expressed as the percentage of rats that ovulated.

### Hormonal replacement with LHRH or EB in non-ovulating rats

A separate group of animals that received micro-injections of atropine in the left POA-AHA were also injected subcutaneously (sc) with 3.7 μg/kg of LHRH (Sigma Chemical Co. St. Louis, MO, USA) at 14:00 h of predicted proestrus day as described [16], or with 10 μg of EB (Sigma Chemical Co.) at 14:00 h on diestrus-2 day as described [17]. All animals were sacrificed on the morning of predicted estrus.

### Measurement of serum hormone levels

A different group animals treated as described above (experiment 1 or 2) was sacrificed at 11:00 or 17:00 h of the expected proestrus day to measure the levels of E_2_, FSH and LH. E_2_ was measured using enzyme-linked immunoassay (ELISA) kits (AccuBind, Monobind Inc., Lake To-est, CA, USA), the assay sensitivity was 6.5 pg/ml. The levels of FSH and LH were measured by radioimmunoassay (RIA) methods with specific reagents and protocols from the National Institute of Diabetes and Digestive and Kidney Diseases (NIDDK), distributed by Dr. A. F. Parlow: LH (NIDDK-HLR-I 125-S-11) and FSH (NIDDK-recombinant FSH-I125-I-9). The intra-assay and inter-assay coefficients of variation for LH and FSH were 12.1 and 14.6 %, respectively, and the sensitivity of the assays for both hormones was 0.1 ng/ml.

### Brain histological procedures

To verify the accuracy of the microinjection site, 100-μm sections of the POA-AHA region were obtained with a vibratome (Technical Products International Inc, St. Louis, MO, USA). The sections were mounted on slides and immediately examined under a stereoscopic microscope. Only results from rats with verified microinjection into the POA-AHA [18].

### Statistical analyses

All statistical analyses were performed with GraphPad InStat3 Software, Inc. (San Diego, CA, USA). Ovulation rate results were analyzed using Fisher’s exact probability test or the Chi-square test. Data on the number of ova shed were analyzed using the Kruskal-Wallis test followed by Dunn’s test. Data on ovarian weight and estradiol, FSH and LH levels were subjected to multivariate analysis of variance (MANOVA) followed by Turkey’s test. All values were expressed as the mean ± S.E.M. A probability value (*p*) ≤ 0.05 % was considered statistically significant.

## Results

### Effects of transient blockade of mAChR in the left or right POA-AHA on spontaneous ovulation

The ovulation rate, number of ova shed and ovarian weight were similar in the vehicle and the intact group. The ovulation rate was lower in animals with atropine injected in the left POA-AHA than in the vehicle group, and it did not change when atropine was injected in the right side. The number of ova shed by animals with atropine injected into the right POA-AHA was lower than in the vehicle group (Table 1). No differences in ovarian weight between groups were observed. Rats with atropine in the left POA-AHA ovulated 48 h after the predicted estrus day (8/8 vs. 2/10, *p* < 0.001).

**Table 1:** Ovulation in rats with unilateral atropine microinjection into POA-AHA performed at 13:00 h of diestrus-2 day

Group	Side of POA-AHA	*n*	Ovulation rate*	Ova shed	
				Left ovary	Right ovary
Intact	–	10	10/10	7.1 ± 0.5	5.4 ± 0.3
Vehicle	Left	9	9/9	6.1 ± 0.9	4.2 ± 1.0
	Right	9	9/9	6.5 ± 0.2	4.8 ± 0.3
Atropine	Left	10	2/10^*a*^	4, 0**	3, 3**
	Right	10	10/10	3.1 ± 0.3^*b*^	1.7 ± 0.2^*b*^

The ovulation rate and the number of ova shed (mean ± SEMs) by rats subjected to microinjection of the vehicle or atropine into the left or right side of the POA-AHA region were measured at the predicted estrus day

^*a*^ *p* < 0.0007 vs. left vehicle (Fisher’s exact probability test)

^*b*^ *p* < 0.001 vs. right vehicle (Kruskall Wallis test, followed Dunn test)

**represents the number of ova shed by the two ovulating rats

*Number of ovulating animals/number of treated animals

Ovulation was induced in 100 % of animals microinjected with atropine in the left POA-AHA that later received injections of synthetic LHRH (atropine + LHRH 5/5 vs. atropine 2/8, *p* < 0.0210, Fisher’s exact test). A similar result was obtained when EB was injected (atropine + EB 5/5 vs. atropine 2/8, *p* ≤ 0.0216).

### Effects of left or right ULO on spontaneous ovulation

On the predicted estrus day, the ovulation rate of the left or right laparotomized groups was similar to that in the intact group. However, the ovulation rate was lower in animals with ULO, compared to the respective laparotomized only groups. The number of ova shed by the remaining ovary (left ovary) in the right ULO group was lower than in the right laparotomized groups. In contrast, no changes were observed between left ULO and left laparotomized rats except for one single rat ovulating in the left ULO group (Table 2). The weight of the ovaries in the right laparotomized animals was higher than those in the intact group. However, the weight of the *in situ* ovaries in the left and right ULO groups were even higher than in the respective laparotomized groups (Table 2). In animals with ULO, vaginal estrus occurred 24 h after ULO. On this day, the animals with left ULO did not ovulate but right ULO rats did.

**Table 2:** Ovulation in rats with unilateral ovariectomy (ULO) performed at 13:00 h of diestrus-2 day

Group	Ovulation rate*	Number of ova shed by the *in situ* ovary		Weight of the *in situ* ovary (mg/100 g bw)***	
		Left ovary	Right ovary	Left ovary	Right ovary
Intact	10/10	7.1 ± 0.5	5.4 ± 0.3	16.5 ± 1.2	15.6 ± 1.2
L-laparotomy	9/10	3.2 ± 1.3^*b*^	4.8 ± 2.6	14.9 ± 1.3	15.6 ± 1.4
L-ULO	1/10^*a*^	–	4**	–	32.7 ± 3.3^*a*^
R-laparotomy	10/10	6.2 ± 1.4	6.0 ± 1.1	24.9 ± 4.0^*b*^	21.7 ± 2.2^*b*^
R-ULO	3/9^*a*^	2.0 ± 0.6^*c*^	–	33.4 ± 3.6^*a*^	–

The ovulation rate and the number of ova shed (mean ± SEMs) and weight of the left or right ovary of intact rats, or rats subject to left (L-laparatomy) or right (R-laparotomy) laparotomy, and those subject to left (L-ULO) or right (R-ULO) ULO were measured at the predicted estrus day

^*a*^ *p* < 0.01 vs. respective laparotomy group (Fisher’s exact probability test)

^*b*^ *p* < 0.01 vs. respective intact group (Kruskall Wallis test, followed Dunn test)

^*c*^ *p* < 0.01 vs. respective laparotomy group (Kruskall Wallis test, followed Dunn test)

***data statistically analyzed by ANOVA followed by Tukey test

**represents the number of ova shed by the ovulating rat

*Number of ovulating animals/number of treated animals

**Fig. 2:**
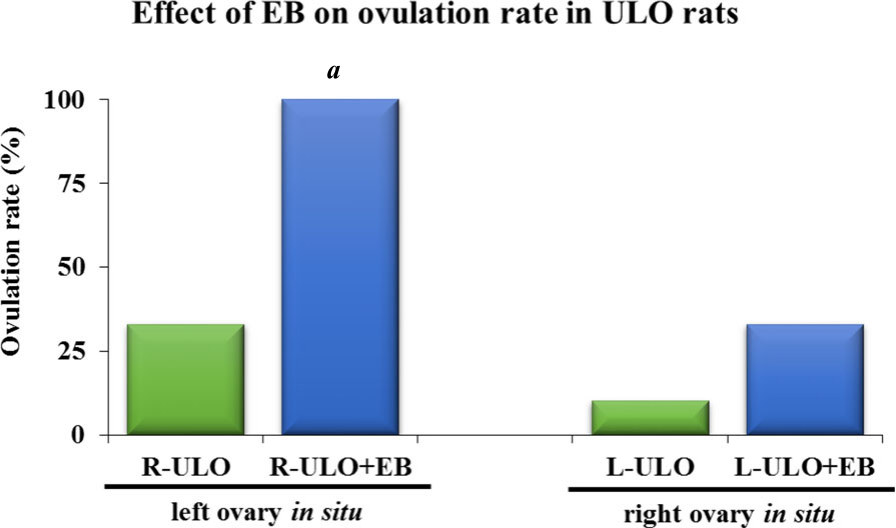
Effects of EB on ovulation in ULO rats. Ovulation rate of the left or right *in situ* ovary of rats subject to left ULO (L-ULO) or right ULO (R-ULO), and ULO rats subsequently injected with 10 μg of EB at 14:00 h on the same day of ULO. The animals were sacrificed at the predicted estrus day. *a* *p* < 0.01 vs. the respective ULO only group (Fisher’s Exact Probability test)

Injection with LHRH re-established ovulation in all rats with ULO (left ULO + LHRH 5/5 vs. left ULO: 1/10; right ULO + LHRH 5/5 vs. right ULO: 3/9, *p* < 0.01 Fisher’s exact probability test). In animals with right ULO (left ovary *in situ*), replacement with EB induced ovulation in all treated animals, while in animals with left ULO (right ovary *in situ*), EB treatment induced ovulation only in 33 % of the treated animals (Fig. 2).

The number of ova shed by animals with right ULO injected with LHRH was higher than in animals with right ULO and no hormone injection (8.8 ± 0.9 vs. 2.0 ± 0.6, *p* < 0.01). LHRH injection in animals with left ULO resulted in higher number of ova shed (7.2 ± 0.4), compared to the single animal with left ULO not treated with LHRH that ovulated (4 ova shed). The number of ova shed by animals with right ULO injected with EB was higher than that of rats with ULO (7.0 ± 0.7 vs. 2.0 ± 0.6, *p* < 0.01). Rats with left ULO and EB injection released a similar number of ova as the group that did not receive hormone treatment (2.3 ± 0.9). The weight of the *in situ* ovaries in animals with left or right ULO and LHRH or EB was lower than in ULO animals without hormonal replacement (Table 3).

**Table 3:** Effects of LHRH or EB on the weight of the *in situ* ovary in rats with unilateral ovariectomy (ULO) performed at 13:00 h of diestrus-2 day

Group	Weight of the *in situ* ovary (mg/100 g bw)	
	Left	Right
L-ULO	–	32.7 ± 3.3
L-ULO + LHRH	–	17.4 ± 1.1^*a*^
L-ULO + EB	–	19.6 ± 1.2^*a*^
R-ULO	33.4 ± 3.6	–
R-ULO + LHRH	19.7 ± 1.5^*a*^	–
R-ULO + EB	18.0 ± 1.3^*a*^	–

The mean ± SEMs of the weight of the left or right ovary of intact rats, or rats subject to left ULO (L-ULO) or right ULO (R-ULO), and ULO rats injected with 3.7 μg/Kg body weight of synthetic LHRH at 14:00 h of the predicted proestrus day, or injected with 10 μg of EB at 14:00 h of the same day of the ULO. The animals were sacrificed at the predicted estrous day

^*a*^ *p* < 0.01 vs. the respective ULO group (MANOVA, followed Tukey test)

### Effects of transitory mAChR blockade in the POA-AHA of ULO rats on spontaneous ovulation

**Fig. 3:**
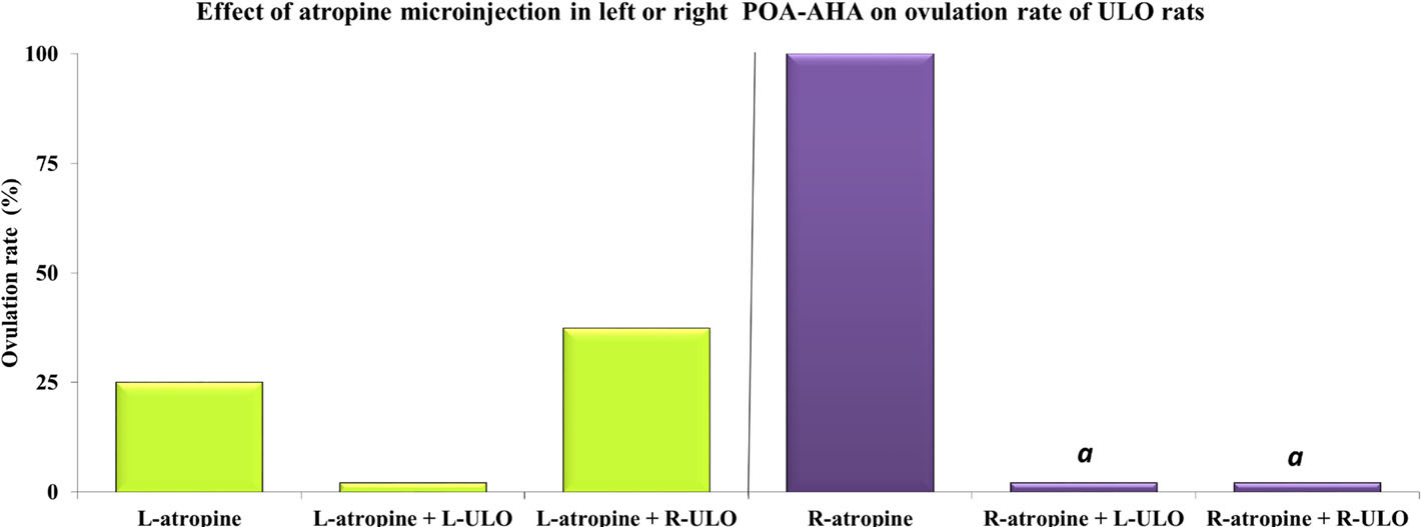
Effects of atropine microinjection into the left (L-atropine) or right (R-atropine) POA-AHA on ovulation in left-ULO (L-ULO) or right-ULO (R-ULO) rats. The animals were sacrificed at the predicted estrus day. *a* *p* < 0.01 vs. R-atropine group (Fisher’s Exact Probability test)

The ovulation rate was not modified in ULO rat microinjected with atropine in the left side of the POA-AHA, while ovulation was blocked by atropine was in the right POA-AHA (Fig. 3). The number of ova shed (data not shown) in ULO rats injected with atropine in either side of the POA-AHA was as low as that in ULO only rats (data not shown).

The weight of the *in situ* ovaries in ULO rats plus atropine injected in either side of the POA-AHA was lower than that in ULO only rats (left ULO + left POA-AHA: 15.0 ± 0.8, left ULO + right POA-AHA: 17.6 ± 0.9 vs. left ULO: 32.7 ± 3.3, *p* < 0.01; right ULO + left POA-AHA: 15.1 ± 0.7, right ULO + right POA-AHA: 17.9 ± 0.4 vs. right ULO: 33.4 ± 3.6, *p* < 0.001).

**Fig. 4:**
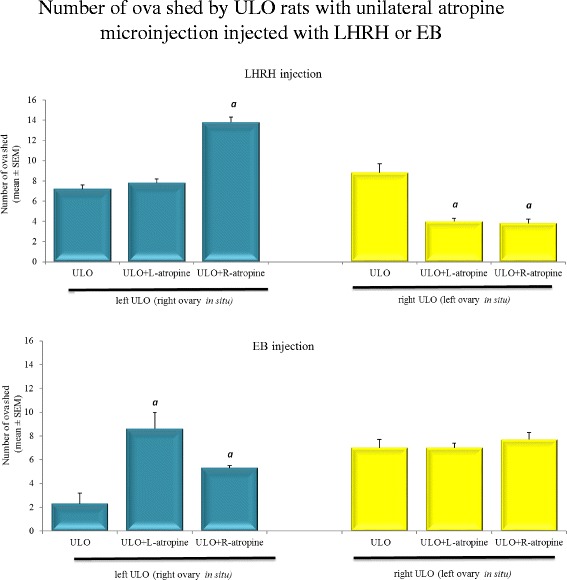
Mean ± SEM of the number of ova shed by ULO rats with unilateral atropine microinjection in the POA-AHA, treated with LHRH or EB. The number of ova shed from the left or right *in situ* ovary of rats subjected to left or right ULO plus atropine microinjection into the left (L-atropine) or right (R-atropine) POA-AHA, performed at 13:00 h of diestrus-2 and sacrificed at predicted estrus day. *a* *p* < 0.01 vs. the respective ULO only group (ANOVA followed Tukey test)

In ULO animals’ microinjected with atropine in either side of the POA-AHA, the number of ova shed induced upon replacement of LHRH or EB, depended on what ovary was remained *in situ*. For example, the injection of LHRH in animals retaining the right ovary *in situ* and microinjected with atropine in the right POA-AHA, shed a higher number of ova than the ULO alone group. By contrast, rats with the left ovary *in situ* microinjected with atropine in either POA-AHA released a smaller number of ova after LHRH injection (Fig. 4).

In ULO animals with the right ovary *in situ*, microinjection with atropine in the right or left POA-AHA and EB replacement resulted in a higher number of ova shed than in the ULO alone group. The injection of EB in rats with the left ovary *in situ* and atropine microinjection in either side of the POA-AHA did not modify the number of ova compared to ULO rats (Fig. 4).

### Effects of transitory mAChR blockade in the left or right POA-AHA on serum hormone levels

At 11:00 h of the predicted proestrus day, the serum levels of E_2_, LH and FSH were similar in all of the experimental groups. However, at 17:00 h animals microinjected with atropine in the left POA-AHA had lower E_2_ and LH serum levels than the vehicle group, while rats treated in the right POA-AHA had levels similar to the vehicle group. FSH levels were higher in animals treated with atropine on the left or right side of the POA-AHA than in vehicle-treated rats (Table 4).

**Table 4:** Effects of atropine microinjected in the left or right POA-AHA on hormone serum levels at 11:00 or 17:00 h on predicted proestrus day

Group POA-AHA	Estradiol pg/mL	LH ng/mL	FSH ng/mL
Proestrus 11:00 h			
Intact	20.3 ± 0.3	0.7 ± 0.3	2.3 ± 0.4
Left vehicle	32.4 ± 9.0	0.4 ± 0.05	1.6 ± 0.13
Left atropine	27.2 ± 5.5	0.2 ± 0.03	2.4 ± 0.8
Right vehicle	33.4 ± 4.9	0.5 ± 0.02	1.2 ± 0.09
Right atropine	21.6 ± 1.9	0.4 ± 0.09	1.2 ± 0.3
Proestrus 17:00 h			
Intact	15.7 ± 2.9	22.8 ± 1.9	7.2 ± 0.50
Left vehicle	23.6 ± 6.0	24.9 ± 2.5	4.9 ± 2.5
Left atropine	3.9 ± 0.9^*abc*^	3.6 ± 1.5^*a*^	98.8 ± 4.4^*abc*^
Right vehicle	37.2 ± 1.3	20.7 ± 1.8	4.1 ± 0.2
Right atropine	33.2 ± 10.2	21.2 ± 2.6	8.3 ± 2.5

The mean ± SEMs for the estradiol, LH and FSH of rats subjected to microinjection of vehicle or atropine into the left or right POA-AHA, at 13:00 h of diestrus-2 day, and sacrificed at 11:00 or 17:00 h on the predicted proestrus day

^*a*^ *p* < 0.01 vs. Left vehicle (ANOVA, followed Tukey test)

^*b*^ *p* < 0.01 vs. right atropine (ANOVA, followed Tukey test)

^*c*^ *p* < 0.01 vs. intact group (ANOVA, followed Tukey test)

### Effects of left or right ULO on hormone serum level measurements

At 11:00 h of the predicted proestrus day, E_2_, LH and FSH serum levels in left ULO rats were higher than those in the left sham group while right ULO did not modify E_2_ levels, but increased LH and FSH levels compared to sham operated rats. At 17:00 h on the predicted proestrus day, LH serum levels in the sham rats were lower than in the intact group. LH and FSH levels in ULO rats were lower than in the sham group (Table 5).

**Table 5:** Effects of ULO performed at 13:00 h on diestrus-2 day on hormone serum levels at 11:00 or 17:00 h on predicted proestrus day

Group	Estradiol pg/mL	LH ng/mL	FSH ng/mL
Proestrus 11:00 h			
Intact	20.3 ± 0.3	0.7 ± 0.30	2.3 ± 0.4
Left laparotomy	14.5 ± 4.0	0.4 ± 0.06	2.0 ± 0.1
Left ULO	61.4 ± 16.1^*ab*^	1.3 ± 0.20^*a*^	42.1 ± 14.7^*ab*^
Right laparotomy	13.0 ± 4.8	0.4 ± 0.01	2.7 ± 0.6
Right ULO	12.4 ± 3.5	2.2 ± 0.40^*a*^	>100^*a*^
Proestrus 17:00 h			
Intact	15.7 ± 2.9	22.8 ± 1.90	7.2 ± 0.50
Left laparotomy	10.8 ± 0.5	12.5 ± 0.01	5.2 ± 0.50
Left ULO	11.5 ± 2.1	0.5 ± 0.10^*a*^	<0.040^*ab*^
Right laparotomy	10.7 ± 1.3	19.4 ± 0.2	8.8 ± 0.8
Right ULO	15.9 ± 3	1.4 ± 0.10^*a*^	2.1 ± 1.04^*a*^

The mean ± SEMs for the estradiol, LH and FSH of rats subjected to left or right laparotomy, and left or right ULO at 13:00 h of diestrus-2 day, and sacrificed at 11:00 or 17:00 h on the predicted proestrus day

^*a*^ *p* < 0.01 vs. corresponding laparotomy (MANOVA, followed Tukey test)

^*b*^ *p* < 0.02 vs. Right ULO (Student “t” test)

## Discussion

In the present study, transient blockade of the mAChRs in the POA-AHA with atropine modified the ovulation process: it inhibited the ovulation rate when the blockade is the left POA-AHA or reduced the number of ova shed when the right POA-AHA was treated with atropine.

We think that the effect of the atropine on right POA-AHA on the number of ova shed but not on ovulation rate may be explained for the lack of muscarinic signals in the POA-AHA to the ovary for the growth and differentiation of follicles. Previously, our group showed that the ovaries of rats with implants of atropine in the POA-AHA had fewer small follicles in the left ovary compared to control group [19].

Regarding the decrease in the levels of the E_2_ and LH on the afternoon of proestrus day after temporary blockade of the muscarinic system of the left POA-AHA, and considering that injection of EB restored ovulation, we believe that these low levels of E_2_ are the cause of the observed blockade of ovulation. These results support the idea that neural signals arising from the left side of the POA-AHA modulate the sensitivity of the ovaries to FSH and LH [19] and by the muscarinc system. In fact, studies performed with subcutaneous injection of atropine on the diestrus-1 day causes a 24-h delay on preovulatory LH and E_2_ levels, and ovulation [20]. Furthermore, the neural information coming from each side of the hypothalamus differentially regulates ovulation in the right and left ovary [19, 21]. Thus, the present study suggests that approximately at noon on diestrus-2 day, the muscarinic system innervating the left POA-AHA regulates in a stimulatory manner the preovulatory secretion of LH, indicating that activation of mAChRs is a key component in stimulating feedback of estrogens and in the pre-ovulatory surge of GnRH.

Tanaka et al. [22] have suggested that peritoneal innervation plays an essential role in the neural connection with the central nervous system. In the current study, the effects of laparotomy on ovarian weight supports that idea because the weight of the ovaries in rats with laparotomy on the right side was greater than in intact animals, possibility meaning that a higher number of follicles were growing. Previous studies by our group showed the participation of the muscarinc system on estradiol secretion in hemiovariectomized rats by laparotomy, these animals displayed an increase of LH and FSH at 11:00 h on the predicted proestrus day suggesting that nerve signal of the ovaries are involved in the regulation of gonadotropin secretion on the proestrus day [23]. Also our group has shown that the removal of the right ovary at 13:00 h on diestrus-2 day resulted in lower E_2_ levels than in rats with perforation in the right side of the dorsal peritoneum, suggesting that it is neural communication with the ovaries what modulates the secretion of E_2_. Based on this previous study and the current results, we postulate that extirpating the left ovary results in the concomitant removal of an inhibitory signal modulating E_2_ secretion by the right ovary [23].

Mechanisms altered by the removal of an ovary are taken over by the remaining ovary. All rats with the left ovary *in situ* (right ULO) ovulated after injection of EB, while the EB had no effect when the right ovary remained *in situ* (left ULO). Despite the different effects on the levels of E_2_ in ULO rats, the levels of FSH and LH increased in the morning of predicted proestrus day and decreased in the afternoon, suggesting that other mechanisms led to increased serum gonadotropin levels. One such mechanism could be down-regulation of E_2_ of estrogen receptors in the hypothalamus and/or pituitary as previously proposed [17, 24]. Classic cholinergic synapses have rarely been observed in GnRHergic neurons, suggesting the presence of a predominantly non-synaptic path in this communication of the cholinergic neuronal system [13]. The current results in rats with muscarinic temporal blockade in the right POA-AHA support previous hypothesis stating that muscarinic neuroendocrine mechanisms regulating ovulation are dependent on the neural information arising from the ovaries and reaching the POA-AHA [12]. It is also likely that a combination of two events is at play here: 1) the absence of estrogen α-receptor in POA-AHA secondary to the blockade of muscarinic receptors as previously shown [25]; and/or 2) a decrease in the number of high affinity sites in the POA and adenohypophysis as suggested [26] as a consequence of changes on hormonal input [23] caused by ULO. Together, these two pieces of evidence allowed us to postulate that acetylcholine mediates, via muscarinic receptors, the feedback effects of gonadal hormones on the secretion of gonadotropins. There is evidence that the ovaries have neural connections with several brainstem nuclei and that representation of the left ovary in the central nervous system is greater than that of the right ovary [27]. In intact 4-day cyclic rats, the left ovary released more eggs than the right [28], and the ability of each ovary to respond to the same endocrine signals was different and variable throughout the estrous cycle [19, 23, 29, 30]. These findings suggest that asymmetric responses of the ovaries to neuroendocrine signals are the result of differences in the innervation received by each ovary and intrinsic differences between them. The current results support this idea because the number of ova shed by the right ovary was higher than that by the left ovary in ULO rats with mAChR blockade that were injected with LHRH or EB.

## Conclusions

Taken together, the present results confirmed that at 13:00 h of diestrus-2 day, activation of mAChRs on the left side of the POA-AHA is required for the preovulatory secretion of GnRH and LH necessary for ovulation. Additionally, stimulation of these receptors is a key component of the stimulatory feedback of estrogens. We propose that the muscarinic system in the right POA-AHA participates in a feedback effect, by steroid hormones, on the preovulatory secretion of gonadotropins and ovulation.

## Abbreviations

ACh: Acetylcholine
E_2_: 17β-estradiol
EB: Estradiol benzoate
FSH: Follicle stimulating hormone
GnRH: Gonadotropin-releasing hormone
LH: Luteinizing hormone
LHRH: Synthetic LH-releasing hormone
mAChRs: Muscarinic receptors
POA-AHA: Preoptic and anterior hypothalamic areas
sc: Subcutaneous
ULO: Unilateral ovariectomy
